# Ambiguities in Sigmoid Volvulus Management: Developing a Framework for Optimal Management Strategies

**DOI:** 10.7759/cureus.98618

**Published:** 2025-12-07

**Authors:** Meghana Taggarsi, Sunetra Chaterjee, Anil Kumar, Karishmah Senthilkumar, Milind Rao

**Affiliations:** 1 Colorectal and General Surgery, Pilgrim Hospital, United Lincolnshire Hospitals NHS Trust, Boston, GBR

**Keywords:** management, mortality, recurrence, sigmoidoscopy, sigmoid volvulus, surgery

## Abstract

Introduction

Sigmoid volvulus (SV) accounts for 10-15% of all large bowel obstructions. Endoscopic decompression remains the first-line management, with a success rate of >80%, but recurrence rates are high (45-71%). To date, ambiguity exists regarding definitive management, particularly when deciding between elective surgery and repeated endoscopic decompression. This study aimed to develop guidelines for the management of SV within our trust.

Methods

This retrospective study was conducted over three years at Pilgrim Hospital, Boston, United Kingdom. Data were collected from online clinical records and case notes. The study was registered with the trust audit department before commencement. Comorbidities were analyzed using the Charlson Comorbidity Index (CCI). Outcome measures included diagnostic modality, success of endoscopic decompression, complications of conservative management, length of hospital stay, definitive surgery, mortality, and recurrence. Descriptive statistics summarized patient demographics, comorbidities, diagnostic tests, and management modalities. Categorical variables are presented as frequencies and percentages, while continuous variables are reported as medians. Kaplan-Meier analysis was used to assess recurrence-free survival from the index admission.

Results

A total of 42 patients with SV were identified, 31 (73.8%) of whom were male. The median age was 77 years. Based on CCI, 32 (76.2%) patients had moderate or severe comorbidities. Twenty-nine patients (69%) received both an abdominal X-ray (AXR) and a CT scan, suggesting limited additional value of AXR, especially since CT is preferred in many centers and is essential for identifying potential complications. Fourteen patients (33%) had previous recurrent SV, including two patients with six prior recurrences. Eighteen (43%) underwent rigid sigmoidoscopy, 12 (28.5%) had flexible sigmoidoscopy, and seven (16.6%) had both, all achieving successful decompression. Four patients (9.5%) experienced spontaneous resolution before endoscopic decompression. Emergency surgery was required in only one patient after failed decompression. The median hospital stay was three days. Fifteen patients (35.7%) experienced further recurrences after the index admission. Overall mortality was two patients (4.8%): one due to perforation after endoscopic decompression and one from perforation secondary to recurrent volvulus, both deemed unfit for surgery. Only five patients (11.9%) underwent definitive surgery, typically after multiple recurrences, with one patient receiving surgery after the 11th recurrence. Kaplan-Meier analysis showed that recurrences commonly occurred within 90 days of the index admission, supporting consideration of early definitive surgery.

Conclusions

Based on this study, we developed local guidelines for expedited management of SV tailored to NHS resources and consistent with World Society of Emergency Surgery recommendations. Optimal management requires a holistic approach integrating early surgical intervention with comorbidity assessment. Early decisions regarding definitive surgery can reduce recurrences, readmissions, morbidity, and healthcare costs.

## Introduction

Colonic volvulus is a critical surgical condition that occurs when a segment of the colon twists around its mesenteric axis [1]. This rotation results in varying degrees of arterial and venous occlusion, as well as partial or complete intestinal obstruction, making it a surgical emergency [2]. The condition predominantly affects the sigmoid colon, accounting for 60-70% of all colonic volvulus cases, followed by cecal volvulus (25-40%) and, more rarely, the splenic flexure or transverse colon (1-4%) [1]. In the United Kingdom and other Western countries, sigmoid volvulus (SV) is most prevalent in elderly males, particularly after the seventh decade of life. In endemic “volvulus belt” regions, including South America, Africa, Eastern Europe, Russia, India, the Middle East, and Brazil, it is more common in males over 40 years of age, with a male-to-female ratio of approximately 4:1 [1].

Risk factors for volvulus are multifactorial. Chronic constipation, frequent laxative use, and anatomic predisposition (long mesentery) are common across all regions. Clinical presentation is typically acute and depends on severity, ranging from features of bowel obstruction to ischemia and perforation. Fulminant cases may present with peritonitis, sepsis, and shock [1,3]. Recommendations suggest a plain abdominal X-ray (AXR) for initial diagnosis, with classic findings such as the coffee bean sign and, occasionally, the “northern exposure sign,” in which the distended sigmoid colon loop extends cephalad beyond the transverse colon [1]. CT of the abdomen and pelvis (CTAP) with contrast is considered the imaging modality of choice when AXR is inconclusive, there is diagnostic uncertainty, or complications are suspected. CTAP may demonstrate a “whirl” sign of the mesentery or a distended sigmoid loop with air-fluid levels [1,4].

Initial management involves fluid resuscitation, urgent blood tests including blood gas and lactate, and endoscopic decompression if there is no suspicion of ischemia or perforation. Endoscopic decompression using rigid or flexible sigmoidoscopy is first-line for acute presentations. Flexible sigmoidoscopy is generally preferred due to superior diagnostic capabilities [1,5]. Although endoscopic decompression has a success rate exceeding 80%, recurrence occurs in 45-71% of patients if definitive management is not pursued [1,5].

Despite SV accounting for 10-15% of large bowel obstructions, ambiguity persists regarding definitive management, particularly when deciding between elective surgery and repeated endoscopic decompression, highlighting the need for further research [1]. To date, there are no published National Institute for Health and Care Excellence (NICE) guidelines on the management of SV or the number of recurrences that should prompt surgical intervention. The World Society of Emergency Surgery (WSES) issued guidelines in 2023, but recommendations were based on low-quality evidence [1]. Sigmoid colectomy has demonstrated the greatest effectiveness in preventing recurrence [1,3,4]. According to NICE guidance, percutaneous endoscopic colostomy (PEC) may be considered for patients with recurrent SV who are unfit for surgery, although its efficacy remains uncertain [6].

Given the lack of local guidance, we conducted a study to assess current practice and develop local guidelines for SV management. The findings were presented as a poster at the Emergency General Surgery Symposium (Association of Surgeons of Great Britain and Ireland, ASGBI) in Manchester in November 2024, under the title “Sigmoid Volvulus-Are We Managing It Right?”.

## Materials and methods

This retrospective cohort study was conducted at Pilgrim Hospital, Boston, one of the two acute sites of United Lincolnshire Teaching Hospitals NHS Trust. A total of 61 patients were admitted with volvulus over a three-year period, from January 2021 to December 2023. The study was registered with the Trust Audit Department prior to commencement, in accordance with trust governance requirements.

Patients aged 18 years and older with SV were included. Of the 61 patients admitted with volvulus, 19 were found to have pseudo-obstruction or volvulus of other parts of the gastrointestinal tract and were therefore excluded. The remaining 42 patients with radiological evidence of SV were included in the analysis.

Case notes, online clinical records, radiological reports, and management plans were reviewed and compiled into a database by multiple reviewers. Data collected included demographics, comorbidities, risk factors, history of recurrence, and management of SV during previous episodes. The dataset was finalized by a senior reviewer prior to analysis.

For the index admission, information was collected on clinical presentation, blood results, diagnostic imaging modality, and both immediate and subsequent management strategies. Patients were stratified according to the Charlson Comorbidity Index (CCI), which quantifies comorbid conditions to assess overall health status and predict clinical outcomes. The CCI evaluates 19 comorbidities, including age, to estimate one-year mortality risk individually or in combination. Individuals with a CCI score of 3-4 have an estimated twofold increase in mortality risk, while those with a score greater than 5 face a fivefold increase [7].

Outcome measures included the mode of management, resolution of volvulus, length of hospital stay, definitive surgical management (emergency or elective), morbidity and mortality following endoscopic procedures, and recurrence after the index admission.

Statistical analysis

The dataset was thoroughly checked for completeness and plausibility. Descriptive statistics were used to summarize patient demographics, comorbidities, diagnostic modalities, and management strategies. Continuous variables are presented as median and range due to a nonparametric distribution. Categorical variables are presented as counts and percentages. Recurrence-free survival following the index admission was assessed using Kaplan-Meier analysis, with time measured from initial diagnosis to first documented recurrence of volvulus. Statistical analysis focused on identifying the timing and pattern of recurrence to provide insights that could guide informed clinical decision-making.

## Results

**Figure 1:**
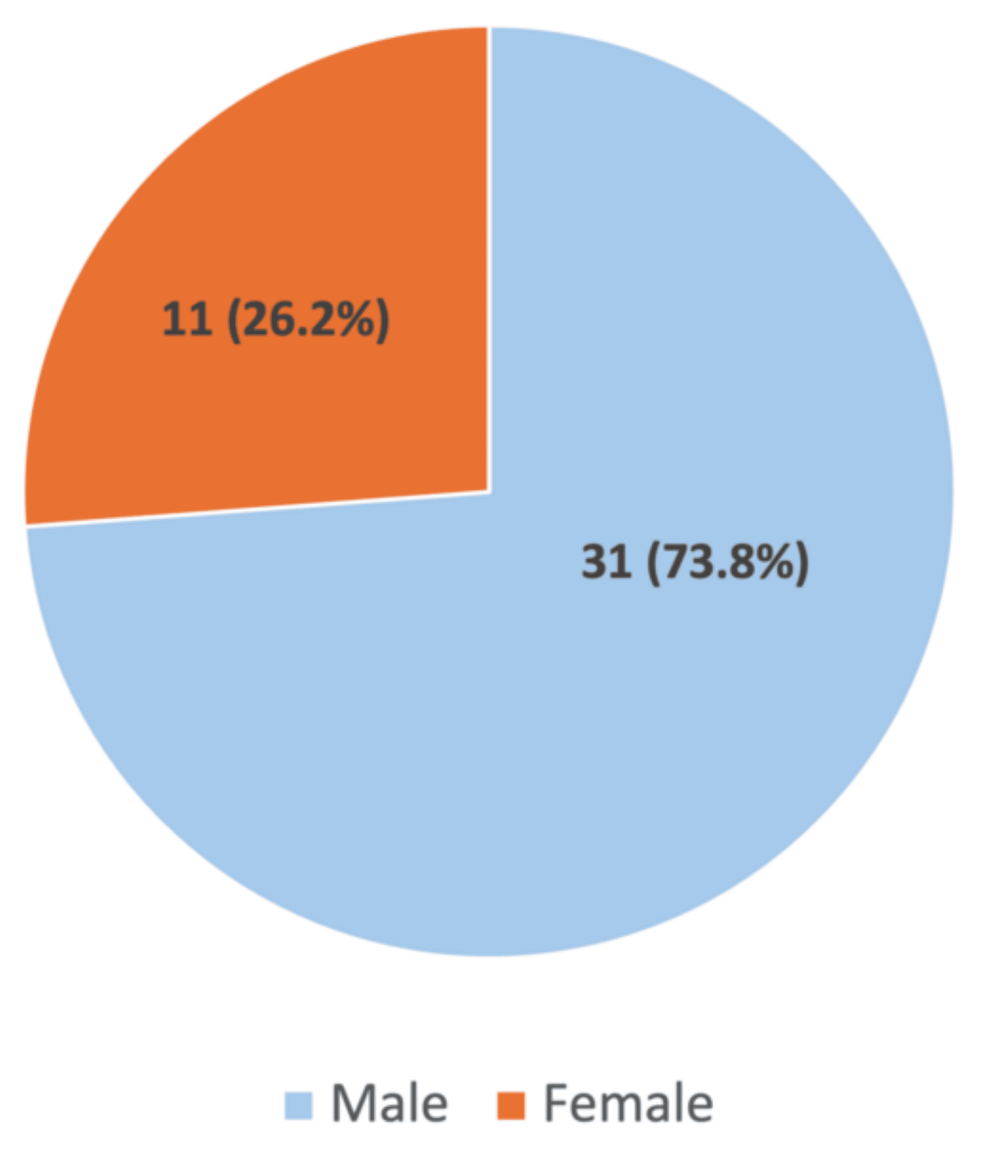
Gender distribution

**Figure 2:**
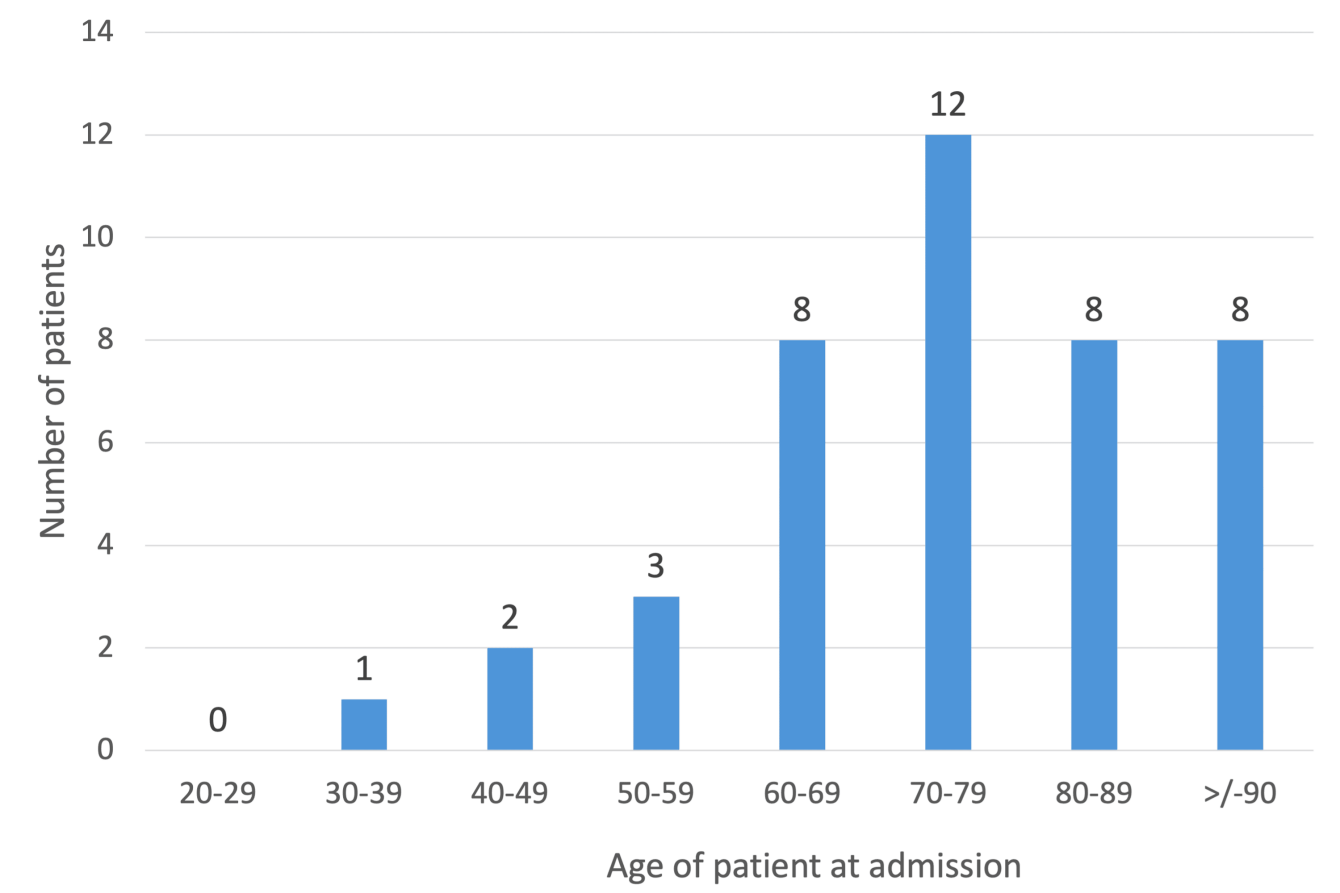
Age distribution at admission

Over the three-year period, 42 patients were admitted with SV. The condition was significantly more prevalent in males, with a male-to-female ratio of 2.8:1, indicating a marked gender disparity (Figure 1). The median age was 77 years (range: 37-98 years) (Figure 2), with 36 patients (85.7%) aged over 60 years.

**Figure 3:**
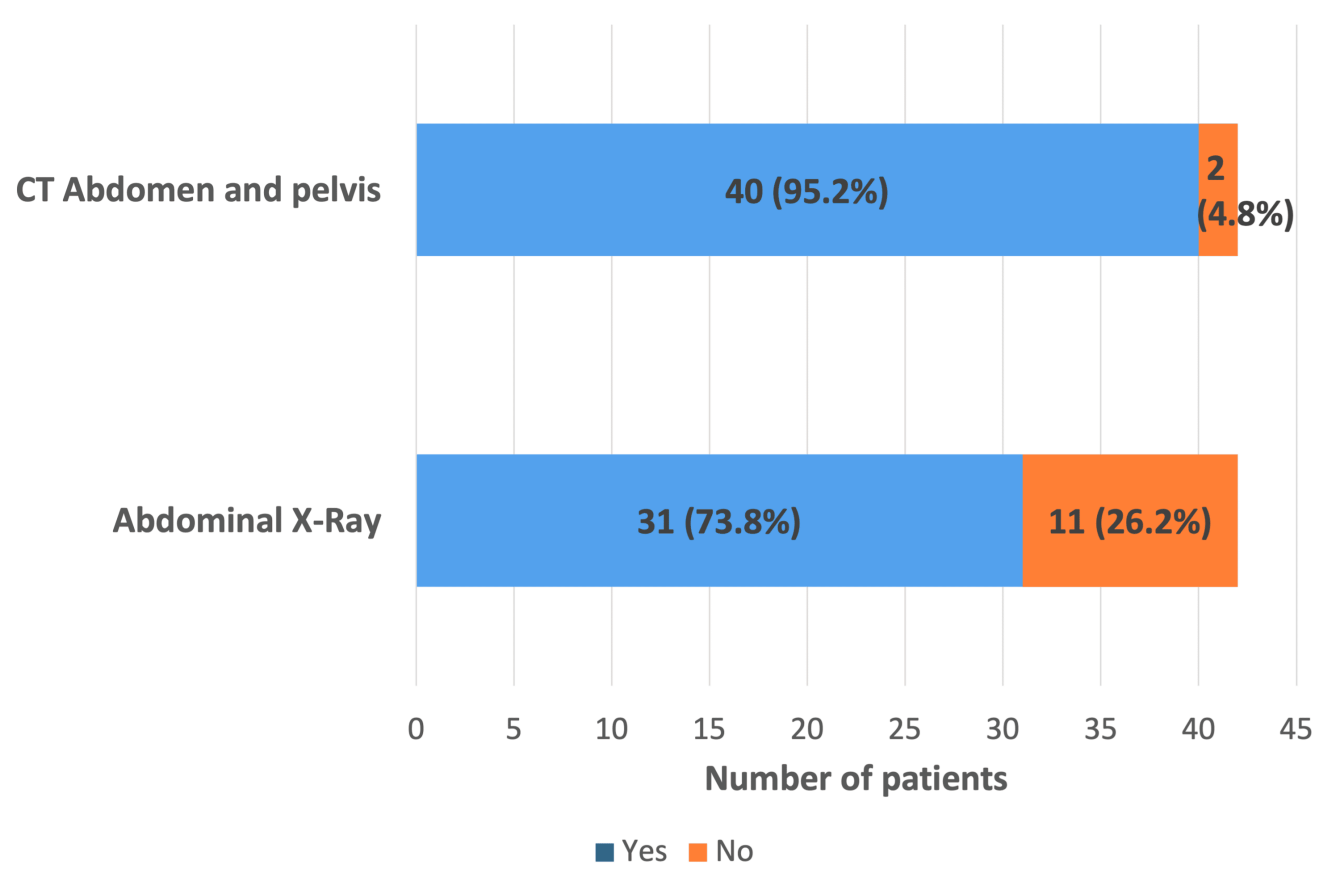
Bar graph illustrating the radiological modalities used for diagnosis

AXR and CTAP were performed for the radiological diagnosis of SV. Thirty-one patients (73.8%) underwent AXR, while 40 patients (95.2%) underwent CTAP. Twenty-nine patients (69%) had both AXR and CTAP for diagnosis (Figure 3).

Twenty-two patients (52.4%) reported regular laxative use. Four patients (9.5%) were care home residents, and eight patients (19%) were bedbound.

**Figure 4:**
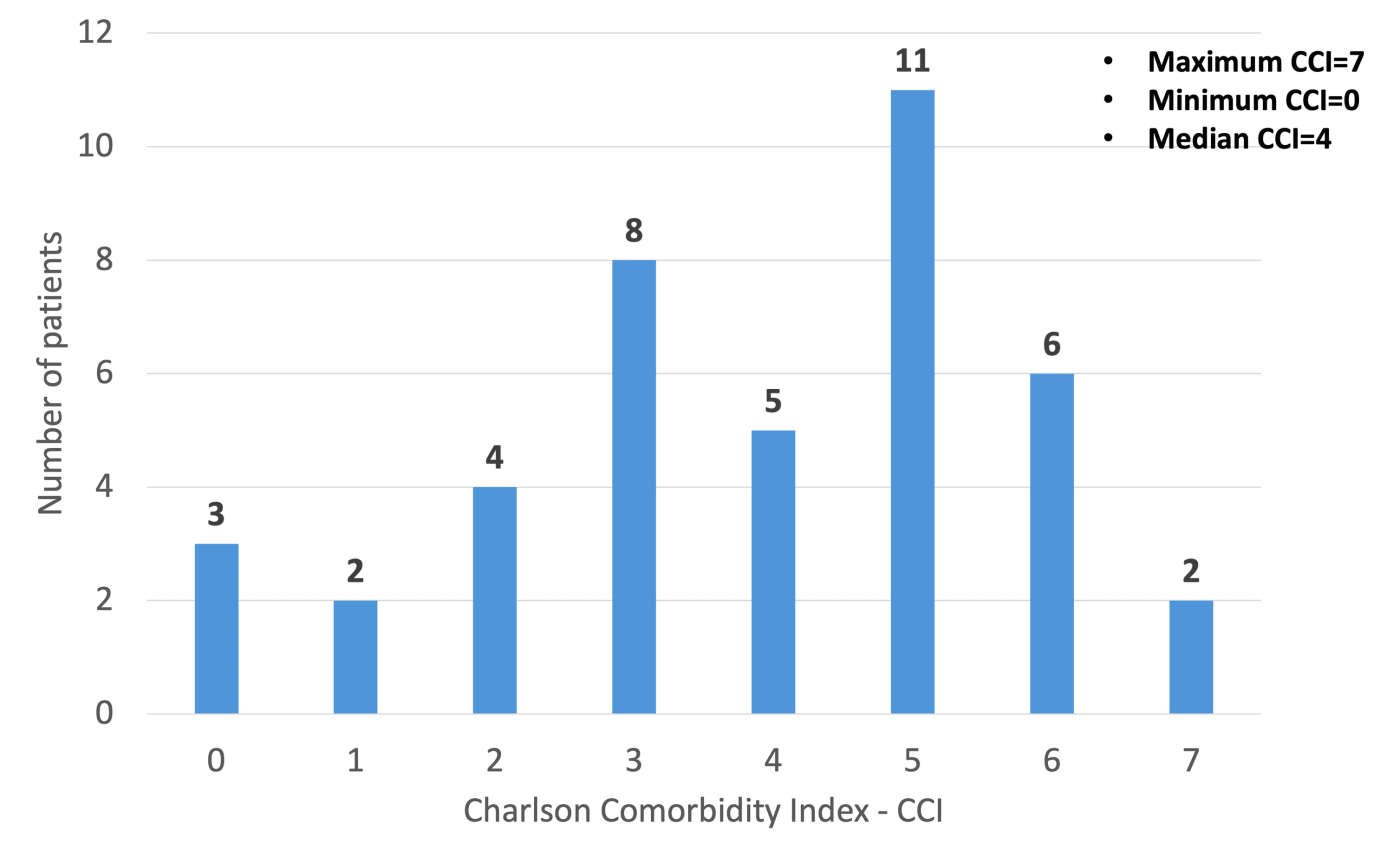
Bar graph illustrating the distribution of patients based on CCI CCI, Charlson Comorbidity Index

Based on the CCI classification, 19 patients (45.2%) had CCI >5, 13 patients (31%) had CCI 3-4, and nine patients (23.8%) had CCI <3. The median CCI was 4, with the highest recorded CCI being 7 (Figure 4). Of the 42 patients, 27 (64.3%) had normal electrolyte levels at presentation. Four patients had elevated lactate, but none correlated with ischemic bowel.

**Figure 5:**
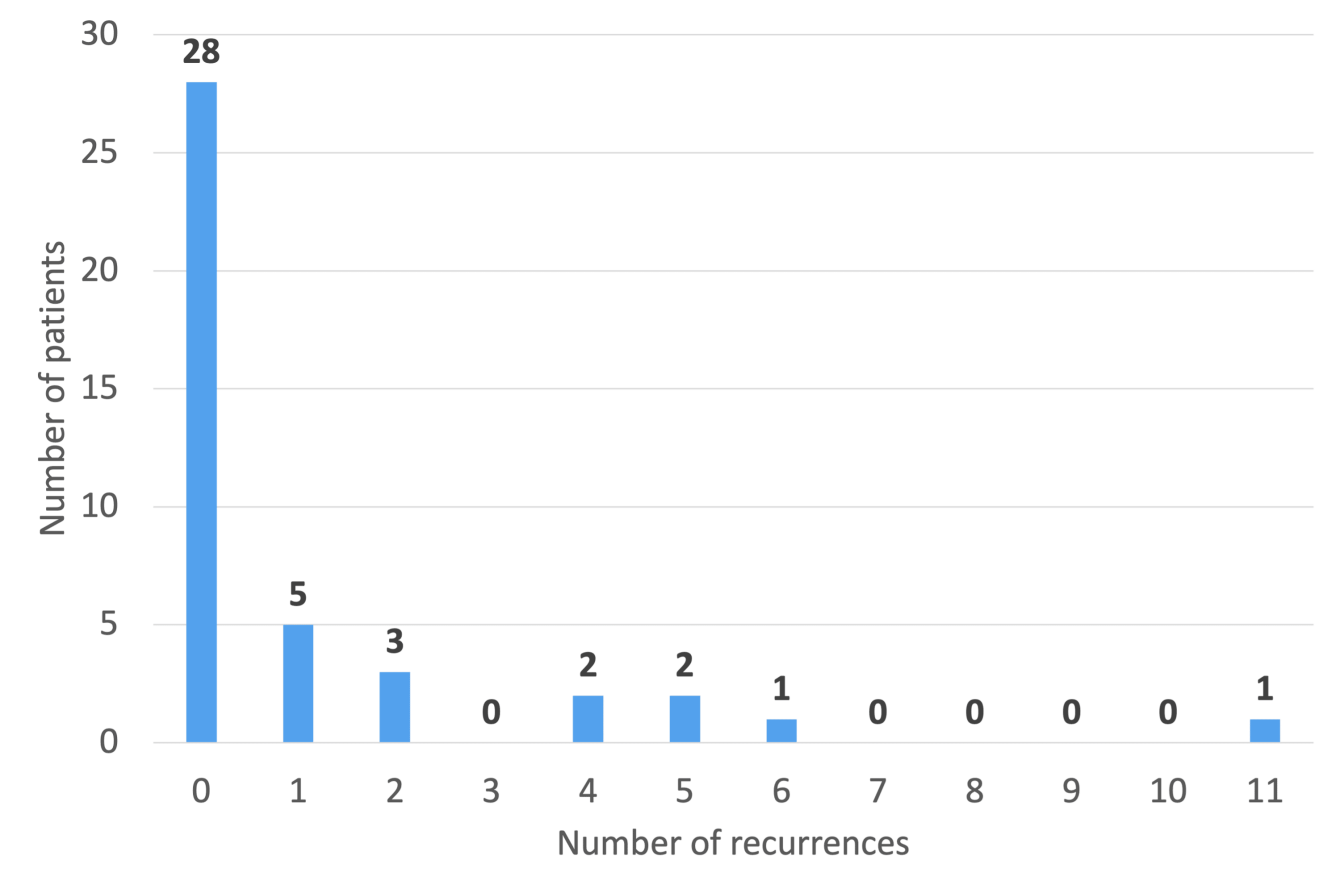
Number of previous recurrences in patients

**Figure 6:**
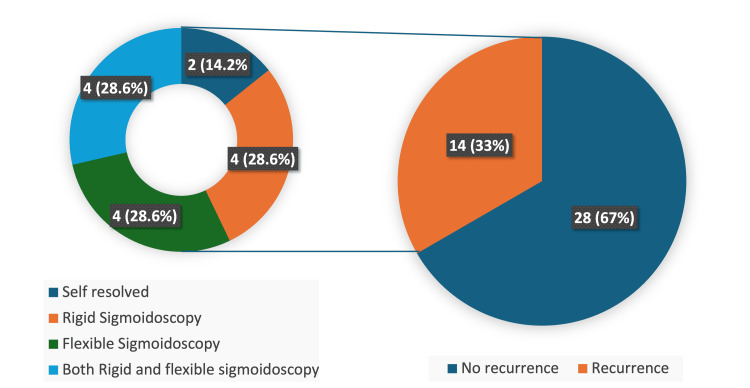
Pie chart representing management of patients during previous admissions

Fourteen patients (33%) had a history of previous recurrent SV before the study period. Among these, two patients had six or more prior recurrences (Figure 5). Regarding prior management, four patients underwent rigid sigmoidoscopy with flatus tube insertion, four underwent flexible sigmoidoscopic decompression, and four underwent both rigid and flexible sigmoidoscopic decompression (Figure 6).

During the index admission, four out of 42 patients experienced spontaneous resolution of volvulus. Eighteen patients (42.8%) underwent decompression using rigid sigmoidoscopy with flatus tube insertion, and 12 patients (28.6%) underwent flexible sigmoidoscopic decompression. Seven patients (16.6%) received both rigid and flexible sigmoidoscopic decompression. One patient had a failed flexible sigmoidoscopic decompression and subsequently underwent a Hartmann’s procedure three days later as definitive management. The overall median length of hospital stay among the 42 patients was three days, with a maximum of 40 days.

Fifteen patients (35.7%) experienced further recurrences after the index admission, with a median time to recurrence of 89 days. One patient was readmitted within nine days and managed conservatively, as he had been deemed unfit for surgery during the index admission. Another patient was readmitted after 75 days with a perforated sigmoid secondary to recurrent volvulus, while a third patient developed a perforation following endoscopic decompression. Both of these patients were frail and unfit for surgical intervention and were therefore managed palliatively; they subsequently died.

**Figure 7:**
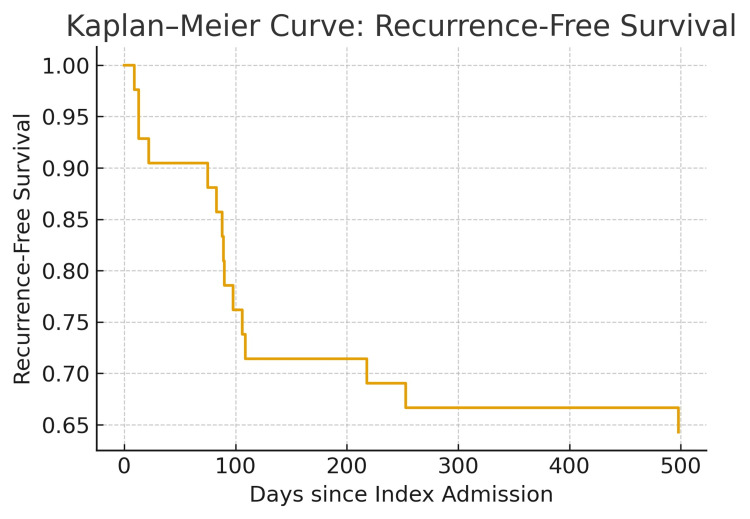
Kaplan-Meier curve demonstrating recurrence-free survival following the index admission for SV The curve shows the proportion of patients who remained free from recurrence over time. A marked early decline is observed within the first 100 days, indicating that most recurrences occurred soon after initial management. Beyond approximately 200 days, the curve plateaus, suggesting that patients who did not experience recurrence within the early months were less likely to recur later. The estimated median time to recurrence was 89 days. Overall recurrence was 35.7% during the follow-up period (January 2021 to December 2023). Censored data points represent patients who did not experience recurrence during the study time frame. SV, sigmoid volvulus

Early recurrence, defined as recurrence within 90 days, occurred in nine of the 15 patients (60%). The Kaplan-Meier curve demonstrated a steep decline in recurrence-free survival during the early period following the index admission, reaching approximately 65-70% at one year, after which the curve plateaued. These findings suggest that patients managed without definitive surgical intervention are at significantly higher risk of early recurrence, particularly those with high frailty and comorbidity burden (Figure 7).

Of the 42 patients, five underwent definitive surgery. Two patients had surgery after their fourth recurrence, one after the fifth recurrence, and another after the sixth recurrence as elective procedures. One patient underwent an emergency Hartmann’s procedure following a failed endoscopic decompression during their 11th recurrence.

## Discussion

This study was performed to assess our single-center experience and to outline local guidelines for evidence-based management of SV at United Lincolnshire Hospitals NHS Trust. As evident from both the literature and our findings, there is a clear lack of standardization in managing SV, which translates into recurrences, readmissions, and excessive use of healthcare resources.

Management of SV involves two components: immediate and definitive interventions. Immediate management includes patient resuscitation, correction of electrolytes and renal function, and endoscopic decompression. Endoscopic detorsion can be performed using rigid or flexible sigmoidoscopy to identify the point of torsion and assess for mucosal ischemia. Flexible sigmoidoscopy has proven to be more effective diagnostically and therapeutically, as rigid sigmoidoscopy can fail to resolve SV and may miss ischemia in up to 24% of cases [1,2,5,8]. In cases presenting with bowel ischemia, perforation, or impending perforation, emergency surgery is indicated.

Given that the presenting population is predominantly elderly, with multiple comorbidities and frailty, a holistic approach is essential. Long-term management and level of care should be considered during the index admission. Since recurrence rates after endoscopic detorsion range from 43% to 75%, patients should be assessed for definitive surgery early, as mortality with emergency presentation can reach 62%. Surgical decisions should involve the patient and family/caregivers, including discussion of potential stoma formation and social support requirements [1,3,5,9]. Recent literature suggests that PEC may reduce recurrences in patients with high operative risk, depending on facility availability [1,6].

In our study, the median age was 77 years, with a male predominance (male-to-female ratio 2.8:1), consistent with published literature [1,8]. SV is more common in institutionalized patients, with other risk factors including dolichosigmoid (elongated sigmoid colon on a narrow mesenteric base), chronic constipation, prior abdominal surgery, pregnancy, diabetes, dementia, and psychiatric disorders such as schizophrenia. In our cohort, four patients (9.5%) were institutionalized, 22 (52.4%) regularly used laxatives, and eight (19%) were bed-bound [1,10-12].

Management decisions and timing of surgery are influenced by performance status and degree of frailty. In our cohort, 32 patients (76.2%) had a CCI indicating moderate (3-4) or high (>5) risk. A recent retrospective study of 78 patients with 332 admissions suggested that early surgery is the optimal approach to recurrent SV, improving quality of life regardless of functional status [1,13]. We recommend that surgical decisions be guided by comprehensive assessment using objective scoring systems, including performance, frailty, and social circumstances.

Our analysis showed that 29 patients (69%) underwent both an AXR and a CTAP scan, suggesting potential redundancy of AXR when CTAP is ultimately performed and highlighting an opportunity to streamline the diagnostic pathway. The classical mesenteric whirl is well visualized on CTAP, which is more sensitive and specific, particularly in the presence of a diagnostic dilemma [1,5,8]. In a retrospective multicenter review by Swenson et al., the diagnostic yield of CTAP was 89%. CTAP is especially useful when perforation or ischemia is suspected, to evaluate for neoplasms, and to differentiate SV from acute colonic pseudo-obstruction (ACPO) [1,14]. ACPO is characterized by massive large-bowel dilatation without mechanical obstruction, arising from autonomic nervous system dysfunction leading to colonic atony. Management is primarily medical, and decompression or surgery is rarely indicated [15].

The cornerstone of SV management is early definitive surgery to prevent recurrence and reduce morbidity and mortality. In our cohort, 14 patients (33%) had prior recurrences and had been managed conservatively or with endoscopic decompression. Emergency surgery was required in only one patient following failed decompression. During index admission, 18 patients (43%) underwent rigid sigmoidoscopy, 12 (28.5%) underwent flexible sigmoidoscopy, and seven (16.6%) underwent both, all with successful decompression. Literature reports endoscopic decompression success rates of 60-95% [1,5,9]. Rigid sigmoidoscopy is a bedside procedure, readily available for emergency admissions in settings like the NHS, and is often accompanied by flatus tube insertion for continued decompression for one to three days [8]. Flexible sigmoidoscopy provides better access to proximal torsion points and allows more comprehensive evaluation of mucosal ischemia. Reported morbidity and mortality rates for rigid and flexible sigmoidoscopy are 3% and 2%, and 0.3% and 1%, respectively [1,5,9]. In our study, one patient (2.3%) experienced a perforation following rigid sigmoidoscopy and was deemed unfit for surgery.

Fifteen patients (35.7%) experienced recurrences after index admission. Recurrence rates after endoscopic decompression range from 43% to 75% [5,8]. Swenson et al. reported a 48% recurrence rate, though the timing of recurrence varies widely [9,14]. In a retrospective study by Slack et al., 74.4% of patients had more than one admission for SV, with a median of three admissions [13].

Kaplan-Meier analysis in our cohort showed that approximately nine patients (60%) recurred within 90 days of index admission, indicating that recurrence risk is greatest shortly after initial management and supporting early definitive surgical intervention in suitable patients. Five patients (11.9%) underwent definitive surgery after ≥4 recurrences, with one patient undergoing surgery after the 11th recurrence. Delays in definitive management led to additional readmissions and morbidity. One patient was readmitted with recurrence and bowel perforation but was unfit for surgery. As shown by Heo et al. and Slack et al., sepsis and elevated CRP levels indicate complicated SV, are associated with a higher likelihood of emergency surgery, and carry a high mortality risk, regardless of surgical intervention. Patients with normal CRP were more likely to undergo successful surgery, whereas elderly, frail patients with sepsis may have poor outcomes [13,16].

Despite extensive literature, there is no consensus on the optimal timing for definitive surgery. The American Society of Colon and Rectal Surgeons recommends definitive surgery during the first episode of SV, during index admission, or soon thereafter, as recurrence risk during index admission is 3-5% [8]. Tsai et al. and Perrot et al. suggested surgery within two to five days post-decompression [17,18]. However, Johansson et al., in a single-center study, found that over 20% of patients did not recur after the first SV episode; recurrences were more common after the second episode (up to 84%), leading to the recommendation that early surgery be considered after the second recurrence to prevent further admissions and reduce healthcare burden [19].

Social circumstances play a critical role in management decisions. Slack et al. analyzed outcomes of SV in relation to a “Social Score” and the level of social support required during recovery. Patients with low social scores benefited more from surgery and had better survival. Frailty alone was not an independent prognostic factor; frail patients requiring extensive social support had poorer outcomes compared to frail patients with adequate support [13]. Although the study did not use a validated social scoring system, it highlights the importance of social factors in decision-making.

Although substantial evidence supports early surgery for better outcomes in SV, current practice is often influenced by surgeon preference, local resource availability, and patient characteristics. SV predominantly affects elderly patients with comorbidities and cognitive impairments, which can limit suitability for major surgery and may affect their ability to understand and retain information.

Clear, evidence-based guidelines regarding surgical indications and timing are urgently needed. Managing this patient group requires a holistic approach that incorporates social care needs after discharge. Early decisions regarding definitive surgery can reduce recurrent admissions and optimize healthcare resource utilization. Comprehensive preoperative assessment, including formal evaluation of social care requirements, should play a central role in guiding management.

Limitations

This was a single-center, retrospective study with a relatively small sample size. While the descriptive nature of the study limits the ability to perform statistical analyses to identify predictors of recurrence, the Kaplan-Meier analysis provides compelling evidence that the highest risk of recurrence occurs within the first 90 days following index admission, strongly supporting the rationale for early definitive management.

**Figure 8:**
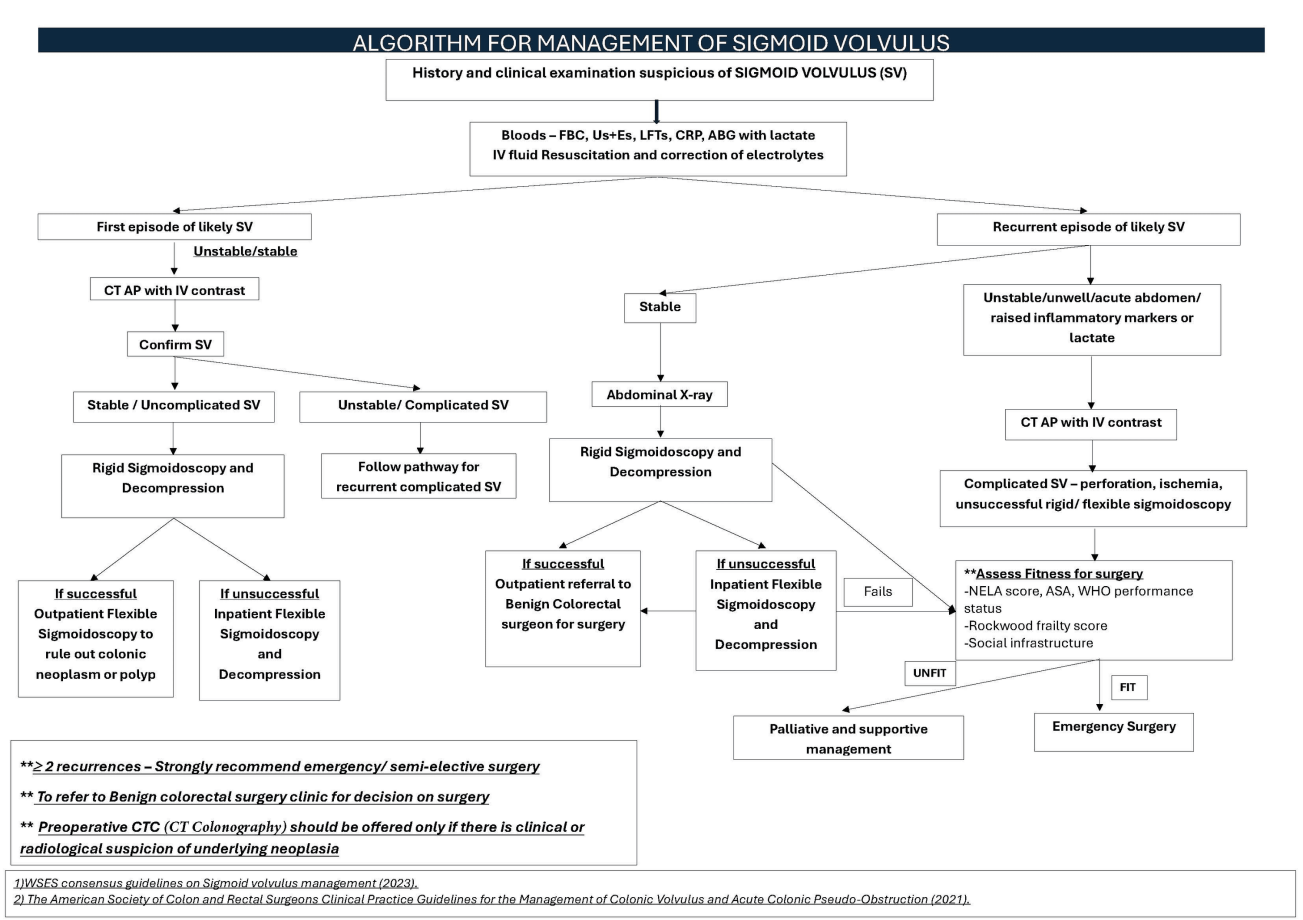
Algorithm for the management of SV ABG, arterial blood gas; AP, abdomen and pelvis; ASA, American Society of Anesthesiologists; CTC, CT colonography; LFTs, liver function tests; NELA, National Emergency Laparotomy Audit; SV, sigmoid volvulus; Us+Es, urea and electrolytes

As a local recommendation, the following algorithm for the diagnosis and management of SV aligns with the WSES guidelines [1,5,8]. This algorithm has been adopted at Pilgrim Hospital as the standard local pathway for patients suspected of having SV (Figure 8).

At our trust, we have also implemented a referral pathway to the Benign Colorectal Surgery team for semi-elective and expedited surgical management to reduce recurrences and readmissions. Additionally, a few NHS trusts have introduced an on-call rota to facilitate out-of-hours emergency flexible sigmoidoscopy for patients with SV.

## Conclusions

This study underscores the ongoing variability in the management of SV and the need for a more unified, patient-centered approach. While endoscopic decompression remains the initial standard of care, our findings suggest that earlier consideration of definitive management is essential, particularly when factoring in clinical status, frailty, social support, and the broader care infrastructure.

A key strength of this work is the development of a pragmatic, locally implemented management algorithm, which provides a clear and streamlined pathway for both initial and recurrent presentations. By integrating clinical assessment, radiological evaluation, endoscopic options, and robust criteria for surgical decision-making, this algorithm offers a practical framework to support consistent care and reduce avoidable recurrences. This represents a meaningful quality improvement tool that could be adapted in other centers facing similar challenges. Future multicenter or prospective studies with larger cohorts, detailed statistical analyses, and structured social-care assessments could further refine this pathway and help establish comprehensive, evidence-based guidelines for the management of SV.

## References

[1] Tian BW, Vigutto G, Tan E (2023). WSES consensus guidelines on sigmoid volvulus management. World J Emerg Surg.

[2] Jones IT, Fazio VW (1989). Colonic volvulus: etiology and management. Dig Dis.

[3] Gingold D, Murrell Z (2012). Management of colonic volvulus. Clin Colon Rectal Surg.

[4] Lieske B, Antunes C (2025). Sigmoid volvulus. StatPearls [Internet].

[5] Atamanalp SS, Disci E, Peksoz R, Atamanalp RS, Atamanalp CT (2023). Management of sigmoid volvulus: a literature review. Ibnosina J Med Biomed Sci.

[6] (2006). Percutaneous endoscopic colostomy. http://www.nice.org.uk/guidance/ipg161.

[7] Charlson ME, Carrozzino D, Guidi J, Patierno C (2022). Charlson Comorbidity Index: a critical review of clinimetric properties. Psychother Psychosom.

[8] Abdelrahim A, Zeidan S, Qulaghassi M, Ali O, Boshnaq M (2022). Dilemma of sigmoid volvulus management. Ann R Coll Surg Engl.

[9] Vogel JD, Feingold DL, Stewart DB, Turner JS, Boutros M, Chun J, Steele SR (2016). Clinical practice guidelines for colon volvulus and acute colonic pseudo-obstruction. Dis Colon Rectum.

[10] Lou Z, Yu ED, Zhang W, Meng RG, Hao LQ, Fu CG (2013). Appropriate treatment of acute sigmoid volvulus in the emergency setting. World J Gastroenterol.

[11] Mulas C, Bruna M, García-Armengol J, Roig JV (2010). Management of colonic volvulus. Experience in 75 patients. Rev Esp Enferm Dig.

[12] Madiba TE, Haffajee MR (2011). Sigmoid colon morphology in the population groups of Durban, South Africa, with special reference to sigmoid volvulus. Clin Anat.

[13] Slack Z, Shams M, Ahmad R (2022). Prognostic factors in the decision-making process for sigmoid volvulus: results of a single-centre retrospective cohort study. BMC Surg.

[14] Swenson BR, Kwaan MR, Burkart NE, Wang Y, Madoff RD, Rothenberger DA, Melton GB (2012). Colonic volvulus: presentation and management in metropolitan Minnesota, United States. Dis Colon Rectum.

[15] Alavi K, Poylin V, Davids JS (2021). The American Society of Colon and Rectal Surgeons clinical practice guidelines for the management of colonic volvulus and acute colonic pseudo-obstruction. Dis Colon Rectum.

[16] Heo S, Kim HJ, Oh BJ (2019). Sigmoid volvulus: identifying patients requiring emergency surgery with the dark torsion knot sign. Eur Radiol.

[17] Tsai MS, Lin MT, Chang KJ, Wang SM, Lee PH. (2006). Optimal interval from decompression to semi-elective operation in sigmoid volvulus. Hepatogastroenterology.

[18] Perrot L, Fohlen A, Alves A, Lubrano J (2016). Management of the colonic volvulus in 2016. J Visc Surg.

[19] Johansson N, Rosemar A, Angenete E (2018). Risk of recurrence of sigmoid volvulus: a single-centre cohort study. Colorectal Dis.

